# SARS‐CoV‐2 and hypertension

**DOI:** 10.14814/phy2.14800

**Published:** 2021-06-13

**Authors:** Briyanth Ravichandran, Daniela Grimm, Marcus Krüger, Sascha Kopp, Manfred Infanger, Markus Wehland

**Affiliations:** ^1^ Department of Biomedicine Aarhus University Aarhus Denmark; ^2^ Department of Microgravity and Translational Regenerative Medicine University Clinic for Plastic, Aesthetic and Hand Surgery Otto‐von‐Guericke University Magdeburg Germany

**Keywords:** COVID‐19, hypertension, MAS‐receptor, renin–angiotensin–aldosterone system, SARS‐CoV‐2

## Abstract

The objective of this review is to give an overview of the pathophysiological effects of the Coronavirus Disease 2019 (COVID‐19) in relation to hypertension (HT), with a focus on the Renin–Angiotensin–Aldosterone System (RAAS) and the MAS receptor. HT is a multifactorial disease and a public health burden, as it is a risk factor for diseases like stroke, coronary artery disease, and heart failure, leading to 10.4 million deaths yearly. Blood pressure is regulated by the RAAS. The system consists of two counter‐regulatory axes: ACE/ANG‐II/AT_1_R and ACE2/ANG‐(1‐7)/MAS. The main regulatory protein in balancing the RAAS is angiotensin‐converting enzyme 2 (ACE2). The protein also functions as the main mediator of endocytosis of the severe acute respiratory syndrome coronavirus 2 (SARS‐CoV‐2) into the host cell. SARS‐CoV‐2 is the cause of COVID‐19 and has caused a worldwide pandemic; however, the treatment and prophylaxis of COVID‐19 are limited. Several drugs and vaccines are currently being tested in clinical trials with a few already approved by EMA and FDA. HT is a major risk factor regarding the severity and fatality of COVID‐19, and the RAAS plays an important role in COVID‐19 infection since SARS‐CoV‐2 can lead to a dysregulation of the system by reducing the ACE2 expression. The exact mechanisms of HT in relation to COVID‐19 remain uncertain, and more research is needed for further elucidation.

## Graphical abstract

This review gives an overview of the pathophysiological effects of the Coronavirus Disease 2019 (COVID‐19) in relation to hypertension, with a focus on the Renin–Angiotensin–Aldosterone–System and the MAS receptor. We summarize the current knowledge about the mechanisms of hypertension in relation to COVID‐19, and the biological processes behind the ACE2 downregulation due to SARS‐CoV‐2.

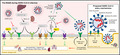

## 1 INTRODUCTION

Hypertension (HT) is a serious public health problem affecting approximately 31.1% of adults worldwide. The disease is multifactorial and a risk factor for cardiovascular diseases, chronic kidney disease, stroke, and premature death (Appel et al., 2003; Mills et al., 2016). The classification of hypertension is given in Table 1.

**TABLE 1:** ISH classification of hypertension (Modified from (Unger et al., 2020))

Category	Office systolic blood pressure (mmHg)		Office diastolic blood pressure (mmHg)
Normal BP	<130	and	<85
High‐normal BP	130–139	and/or	85–89
Grade 1 HT	140–159	and/or	90–99
Grade 2 HT	≥160	and/or	≥100

The renin–angiotensin–aldosterone system (RAAS) plays an important role in regulating blood pressure (BP). BP regulation by the RAAS depends on the interplay of its two axes: angiotensin‐converting enzyme/angiotensin‐II/angiotensin type‐I receptor (ACE/ANG‐II/AT_1_R) and angiotensin‐converting enzyme 2/angiotensin‐(1‐7)/MAS‐receptor (ACE2/ANG‐(1‐7)/MAS). The ACE2/ANG‐(1–7)/MAS‐axis plays a protective role in several diseases, including HT (Azushima et al., 2020).

The novel severe acute respiratory syndrome coronavirus 2 (SARS‐CoV‐2) is the cause of a worldwide pandemic. The virus appeared for the first time in Wuhan in China in 2019 and had been spreading ever since. The virus causes Coronavirus Disease 2019 (COVID‐19) and Patients with COVID‐19 exhibit various symptoms, such as among others fever and cough, but the disease can also lead to respiratory failure and death (Ludwig & Zarbock, 2020). Table 2 summarises several of known symptoms. As of 03/08/2021, 2.594.064 deaths related to COVID‐19 were reported globally (Johns Hopkins University Coronavirus Resource Center, JHU, 2021). SARS‐CoV‐2 gains viral entry by interacting with membrane‐bound angiotensin‐converting‐enzyme‐2 (ACE2). The interaction may affect the expression of ACE2, which plays a key role in balancing the two axes of RAAS. (Azushima et al., 2020; Hoffmann et al., 2020; Li, He, et al., 2020).

**TABLE 2:** Prevalence of common symptoms of COVID‐19

Study	Number of participants	Symptoms and prevalence (%)
(Du et al., 2020)	85	Fever (91.8) Dry cough (22.4) Dyspnea (70.6) Myalgia (16.5) Diarrhoea (18.0)
(Wang, Yin, et al., 2020)	107	Fever (97.2) Dry cough (62.6) Dyspnea (32.7) Fatigue (64.5) Myalgia (30.8) Diarrhoea (6.5) Nausea (5.6)
(Guan et al., 2020)	1099	Cough (67.8) Dyspnea (18.7) Fatigue (38.1) Myalgia or arthralgia (14.9) Headache (13.6) Sputum production (33.7)
(Agyeman et al., 2020) (meta‐analysis)	Olfactory dysfunction: 24 studies, 8435 participants in total. Gustatory dysfunction: 15 studies, 5649 participants in total.	Olfactory dysfunction: Pooled prevalence (41.0 (28.5–53.9)) Gustatory dysfunction: Pooled prevalence (38.2 (24.0–53.6))

The objective of this review is to summarise the pathophysiological effects of COVID‐19 in relation to hypertension and with focus on the RAAS and the MAS receptor.

## 2 METHODS

The literature used for this review was mainly accessed through www.Pubmed.gov. Search terms were combined to limit the number of results. By filtering the results by year and only considering results starting from 2015, the number of hits were further decreased. Additionally, the number of results could be further reduced by searching for specific types of articles such as clinical studies, meta‐analyses and clinical trials.

‘Hypertension AND COVID‐19’ gave 56 results when filtering for meta‐analysis’. ‘COVID AND RAAS’ gave 107 results. ‘RAAS AND ACE2’ gave 116 results. ‘2019‐ncov AND structure’ gave 120 results when filtering for clinical studies. ‘(RAAS +MAS receptor) OR (renin–angiotensin–aldosterone system +MAS receptor)’ gave 426 results. ‘COVID‐19 AND vaccine’ gave 36 results when filtering for clinical studies. Another way of finding articles was using the function ‘Similar articles’ in www. Pubmed.gov, and using the reference lists of other articles.

Aside from www.Pubmed.gov, literature about treatment of hypertension, treatment of COVID‐19 and routes of transmission were accessed through articles from www.who.int, and using the reference lists of the articles. Furthermore, some information about vaccines and drug candidates were accessed through www.ema.europa.eu.

## 3 HYPERTENSION

Hypertension is a condition with a persistent elevated systolic (≥140 mmHg) (SBP) and/or diastolic blood pressure (DBP) (≥90 mmHg). Depending on the guidelines issued by the various societies, both the definition of hypertension and as the target blood pressure under therapy can vary. In 2020, the International Society of Hypertension has extracted the evidence‐based content from multiple recent guidelines and proposed standards for worldwide practice, which can be applied both under low‐ as well as high‐resource conditions. In general, BP is classified into four categories, as illustrated in Table 1 (Unger et al., 2020). According to the American Heart Association, HT is the main cause of death globally and compromises about 10.4 million people yearly. HT is a serious public health problem because it is a common risk factor for cardiovascular diseases like coronary artery disease (accounting for 25–30% of acute myocardial infarctions), stroke and heart failure (Unger et al., 2020). The disease is multifactorial, involving genetic and environmental factors such as smoking and diet (Rossier et al., 2017). There are two types of HT, primary and secondary HT. They are defined as HT with no clear underlying cause and HT with an underlying cause, respectively. 5–10% of HT patients have a secondary HT (Unger et al., 2020).

The treatment of HT includes lifestyle modifications such as weight reduction and regular physical activity. Studies have shown that these behavioural changes lower BP and reduce the risk of cardiovascular diseases (Appel et al., 2003; Bacon et al., 2004; Unger et al., 2020). Patients with grade 1 and grade 2 HT are treated with antihypertensive drugs (Unger et al., 2020).

The classes of blood pressure medications include among others AT_1_‐Receptor antagonists (ARBs), ACE inhibitors (ACEIs), calcium channel blockers (CCBs), thiazides/thiazide‐like diuretics, beta‐adrenoreceptor antagonists (BAAs), α‐adrenoreceptor antagonists, centrally active alpha‐adrenergic agonists, alpha‐2 receptor agonists and combined alpha‐ and beta‐adrenoreceptor antagonists.

First line drugs are RAAS blockers, CCBs and diuretics. Depending on the severity of HT, the drugs can be applied as mono‐ or combination therapy. The combination depends on the comorbidities or complication(s). Most patients with grade 1 or 2 HT require combination therapy (Unger et al., 2020; Williams et al., 2018).

## 4 THE RENIN–ANGIOTENSIN–ALDOSTERONE SYSTEM AND THE MAS RECEPTOR

The RAAS is responsible for the regulation of BP, electrolyte balance and extracellular fluid. Dysregulation of the RAAS can lead to HT (Azushima et al., 2020; Patel et al., 2017). In response to a low BP, low NaCl and activity of the sympathetic nervous system, the renal juxtaglomerular cells release renin. This protein catalyses the formation of angiotensin I (ANG‐I) from angiotensinogen, which is secreted from the liver. ANG‐I is then cleaved by angiotensin‐converting enzyme (ACE) producing angiotensin II (ANG‐II). ANG‐II binds to the ANG II type 1 receptor (AT_1_R) in various tissues in the body. The effects of the receptor activation are vasoconstriction, stimulation of aldosterone secretion, and stimulation of the thirst reflex leading to secretion of antidiuretic hormone (ADH). The released aldosterone stimulates the sodium and water reabsorption in the renal distal tubules and collecting ducts. ADH binds to receptors in the renal collecting ducts and decreases urinary loss. The effects of ANG‐II, aldosterone and ADH increase cardiac pre‐ and afterload, leading to an increase in BP. Furthermore, the activation of the ACE/ANG‐II/AT_1_R axis is linked to oxidative stress, fibrosis, and inflammation (Booth et al., 2002; Patel et al., 2017; Santos et al., 2019).

ANG‐II also binds to the ANG II type 2 receptor (AT_2_R). The effects of this interaction are the opposite of ANG‐II activation of AT_1_R: antifibrosis, vasodilation, and anti‐inflammation (Azushima et al., 2020).

**FIGURE 1:**
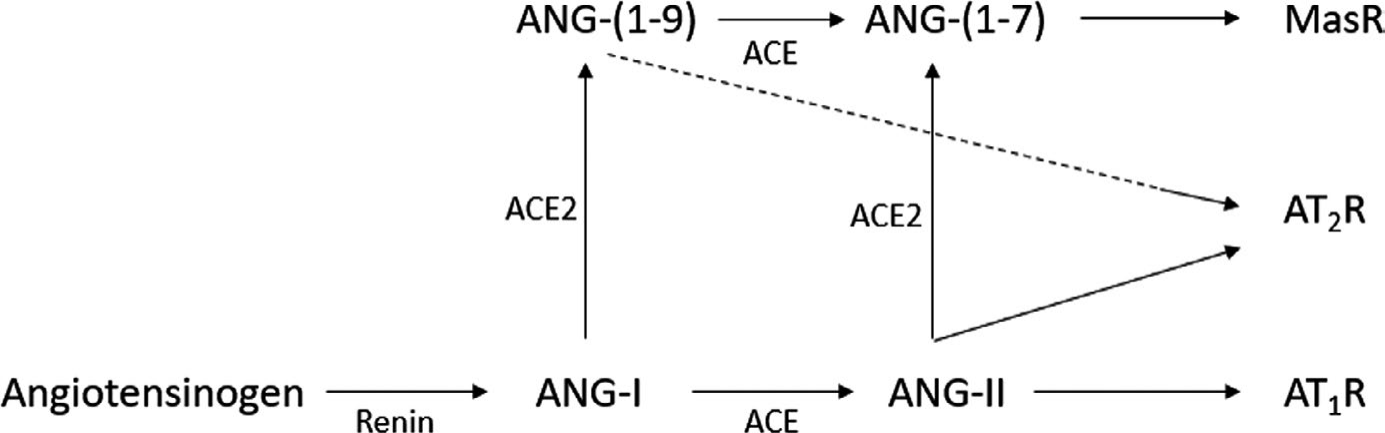
Overview of the RAAS. ANG‐I, angiotensin I; ANG‐II, angiotensin II; ANG‐(1‐7), angiotensin‐(1‐7); ANG‐(1‐9), Angiotensin‐(1‐9); ACE, angiotensin converting enzyme; ACE2, angiotensin converting enzyme 2; AT1R, angiotensin type‐I receptor; AT2R, angiotensin type‐II receptor, MasR, MAS receptor

The ACE2/ANG‐(1‐7)/Mas axis is a counter‐regulatory system of the ACE/ANG‐II/AT_1_R axis; Figure 1 illustrates the two RAAS axes. The main regulatory enzyme in balancing the RAAS is ACE2, a membrane‐bound monocarboxypeptidase expressed in cardiovascular tissue, the kidneys, lungs, liver, small intestine, and brain (Santos et al., 2019). It converts ANG‐I to ANG‐(1‐9), but more importantly, it converts ANG‐II to ANG‐(1‐7). Apart from having an affinity to the AT_2_R, ANG‐(1‐9) is cleaved by ACE producing ANG‐(1‐7). Thus, ACE2 reduces the amount of the substrate for ACE and the amount of circulating ANG‐II, limiting the effects of the ACE/ANG‐II/AT_1_R axis. ANG‐(1‐7) has a high affinity to the MAS receptor (MasR). The MasR is a G protein‐coupled receptor. In humans it is mainly expressed in enterocytes, renal tubules, gallbladder, cardiomyocytes, male reproductive cells, placental trophoblasts, ductal cells, eye, and vasculature (Hikmet et al., 2020), and its activation leads to various effects such as antiarrhythmic effects and vasodilation through the release of nitric oxide (NO) and prostaglandins (Flores‐Muñoz et al., 2011; Povlsen et al., 2020; Santos et al., 2013). Furthermore, studies on mice have shown that ANG‐(1‐7) reduces oxidative stress and vascular inflammation. Hammer et al. observed that a deletion of the MasR induced a proinflammatory phenotype of macrophages in mice (Hammer et al., 2016). In addition, Shenoy et al. observed that the ACE2/ANG‐(1‐7)/MAS axis is protecting the lungs from fibrosis and pulmonary hypertension in rats (Shenoy et al., 2010). These studies indicate that the ACE2/ANG‐(1‐7)/MAS axis has an anti‐inflammatory and anti‐fibrotic effect.

## 5 SEVERE ACUTE RESPIRATORY SYNDROME CORONAVIRUS 2

**FIGURE 2:**
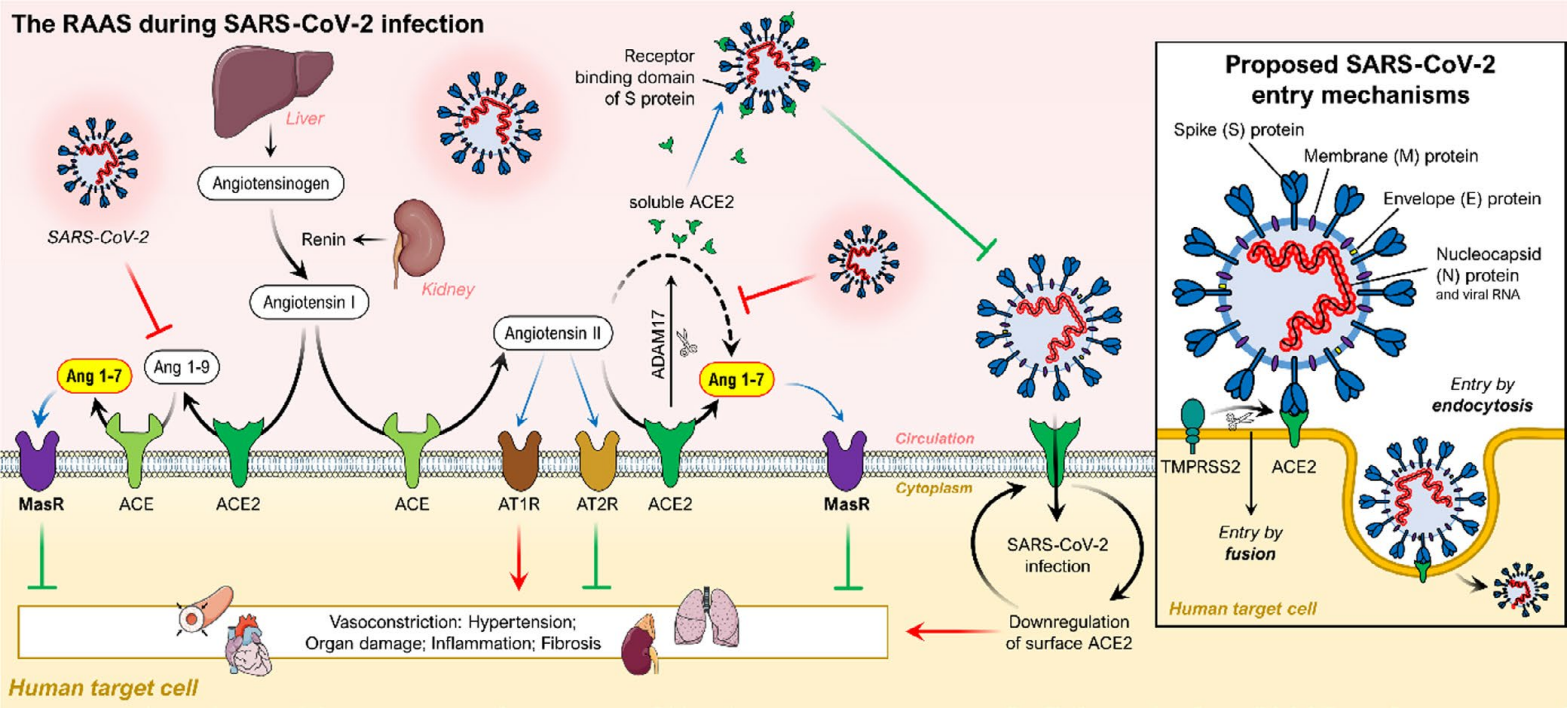
Schematic diagram of the RAAS, its protective effects on organs and its interactions with the SARS‐CoV‐2 viral entry mechanism. The protease renin cleaves angiotensinogen to generate angiotensin I. ACE plays an important role in converting angiotensin I into angiotensin II. Angiotensin II may exert some biological functions through angiotensin II receptor type 1 and 2 receptors (AT1R and AT2R), leading to potent vasoconstriction in several organs. ACE2 hydrolyses angiotensin II to the Angiotensin 1‐7, which binds the Mas receptor playing a protective role in several organs. ACE2 also hydrolyses angiotensin I to the Angiotensin 1‐9, which can be further converted to Angiotensin 1‐7 by ACE. The balance between ACE/Ang II/AT1R and ACE2/Ang 1‐7/MasR axes is a prerequisite for maintaining normal health. In addition, ADAM17 can cleave the extracellular juxta‐membrane region of ACE2. Soluble ACE2 can bind to the receptor‐binding domain of the spike protein of SARS‐CoV‐2 thus blocking further binding to ACE2 and host cell infection. (Small insert) Illustration of the viral entry of SARS‐CoV‐2. The receptor‐binding domain of the spike protein from SARS‐CoV‐2 binds to ACE2, allowing host cell entry and infection. TMPRSS2: transmembrane protease serine 2. Adapted from (Abd El‐Aziz et al. 2020)

The novel SARS‐CoV‐2 is a spherical enveloped positive‐strand RNA virus and a member of the β‐coronavirus group (Zhu et al., 2020). It has a strong resemblance to SARS‐CoV, sharing 80% of sequence identity and employing similar viral entry mechanisms (Chen et al., 2020). However, SARS‐CoV‐2 has a significantly higher binding affinity to ACE2, and thus, the number of virus particles required for infecting a cell is lower (Shang et al., 2020). The genome of SARS‐CoV‐2 codes for viral structural proteins such as spike (S) glycoprotein, envelope (E), membrane (M) and nucleocapsid (N). Especially the S protein plays a vital role in viral entry to the host cells, as illustrated in Figure 2 (Woo et al., 2010).

The interaction between the S protein, the serine protease transmembrane serine protease 2 (TMPRSS2) and membrane‐bound ACE2 mediates the endocytosis of the virus into the cell. The S protein is a class I fusion protein and contains an S1 and S2 subunit. The S1 subunit mediates the attachment to membrane‐bound ACE2. TMPRSS2 is co‐expressed with ACE2 on type II pneumocytes. The enzyme induces conformational changes in the S2 subunit by cleavage. The conformational changes activate the subunit facilitating the membrane fusion into the host cell (Glowacka et al., 2011; Hoffmann et al., 2020; Wang, Zhang, Wu, et al., 2020; Zou et al., 2020). Apart from ACE2, alternative mechanisms of entry of the SARS‐CoV‐2 into the host cells are through interaction with AT_2_R and CD147. It has been shown that the AT_2_R has an affinity for the S protein and interacts with ACE2 and thus indicating the receptor playing a role in the viral entry into the host cells (Cui et al., 2020). In addition, the glycoprotein CD147 has been shown to interact with the S protein and facilitates viral entry to host cells suggesting CD147 as another possible receptor for viral entry of SARS‐CoV‐2 (Wang, Chen, et al., 2020).

Following the endocytosis, the nucleocapsid is released into the cell where replication and translation of the genome occurs. The translated RNA and proteins are assembled in the endoplasmic reticulum and Golgi apparatus, after which the viruses are released from the cell by exocytosis (Hussain et al., 2020).

The virus has a median incubation period of approximately 5 days, but it can last up to 14 days (Lauer et al., 2020). SARS‐CoV‐2 is transmitted from human to human (Li, Guan, et al., 2020). There have been cases with SARS‐CoV‐2 transmission from humans to animals such as dogs, cats, tigers, monkeys and minks. However, it is not clear if the virus can be transmitted back to humans again (Oreshkova et al., 2020; Sit et al., 2020). The main routes of transmission are through physical contact, respiratory droplets, fomites, and aerosols. The virus is capable of remaining infectious in aerosols and on surfaces for hours and days, respectively (van Doremalen et al., 2020; Li, Guan, et al., 2020; Tang et al., 2020). Some studies suggest that the virus may be transmitted faecal‐orally (Chen, Chen, et al., 2020; Ong et al., 2020). There have also been cases indicating vertical transmission of the virus (Sisman et al., 2020; Vivanti et al., 2020).

The carriers of SARS‐CoV‐2 can be either symptomatic, presymptomatic or asymptomatic (Furukawa et al., 2020; Li, Ji, et al., 2020; Rothe et al., 2020; Tong et al., 2020). Most patients infected with SARS‐CoV‐2 present mild symptoms such as fever and cough. Other symptoms that have been observed are malaise, dyspnoea, myalgia, sputum, fatigue, sore throat, and nausea (Du et al., 2020; Guan et al., 2020; da Rosa Mesquita et al., 2020; Wang, Yin, et al., 2020). In addition, a high prevalence of reversible olfactory and gustatory dysfunction was reported (Agyeman et al., 2020). Table 2 illustrates the prevalence of common symptoms of COVID‐19.

As a sign of worse disease progression, patients show leukopenia and lymphopenia (Guan et al., 2020; Yue et al., 2020). Chest computed tomography scans (CTs) of some patients with SARS‐CoV‐2 infection show bilateral pneumonia (Du et al., 2020; Guan et al., 2020; Wang, Yin, et al., 2020; Yue et al., 2020). Pneumonia is a common clinical manifestation of the COVID‐19 and can lead to acute respiratory distress syndrome (ARDS). Other common complications are shock, acute kidney injury (AKI) and acute cardiac injury, with ARDS being the most common cause of death (Hussain et al., 2020; Wang, Yin, et al., 2020). Studies have also shown an increase in embolic events (pulmonary embolism, venous thromboembolic events, deep vein thrombosis) in patients with COVID‐19 (Lu et al., 2020). Additionally, there have been cases with myocarditis, myopericarditis, acute haemorrhagic colitis and placental inflammation (Carvalho et al., 2020; Craver et al., 2020; Hosier et al., 2020; Kim et al., 2020; Naneishvili et al., 2020). Many patients that develop severe complications have comorbidities of HT, diabetes, or cardiovascular disease, indicating that these diseases might be risk factors for developing severe clinical manifestations (Du et al., 2020; Wang, Yin, et al., 2020; Zhou et al., 2020). Another significant risk factor for severe COVID‐19 is an age over 65 years. Studies by Zhou et al. and Du et al. showed that the average age of nonsurvivors was 69 years, and 61% of 85 fatal cases were ≥65 years, respectively (Du et al., 2020; Zhou et al., 2020). Additionally, a meta‐analysis by Zheng et al. estimated that patients with an age of >65 years have a 6‐fold (OR = 6.01 (3.95, 9.16), *p* < 0.00001) increased risk of severe COVID‐19 (Zheng et al., 2020). So far, approximately 2.593 million deaths related to COVID‐19 have been reported (Johns Hopkins University Coronavirus Resource Center, March 8, 2021).

The science regarding treatment of COVID‐19 is fast‐changing. Mainly symptomatic treatment is recommended by the WHO (WHO, 2020). Drugs such as hydroxychloroquine and remdesivir have been tested as treatments for patients with COVID‐19. Remdesivir has been shown to have clinical benefits in relation to COVID‐19 treatment, but a study has also found no significant benefits of the treatment. Also, hydroxychloroquine has shown not to be associated with clinical benefits (Beigel et al., 2020; Singh et al., 2020; Wang, Zhang, Du, et al., 2020). According to results from WHO’s ongoing Solidarity Trial the antiviral therapies remdesivir, hydroxychloroquine, lopinavir/ritonavir and interferon had little or no effect on overall mortality in hospitalized patients with COVID‐19, and only corticosteroids have been proven as an effective treatment against severe COVID‐19 (Pan et al. 2020; The RECOVERY Collaborative Group (2021)). EMA has approved the use of the glucocorticoid dexamethasone for treatment of COVID‐19 patients on oxygen or mechanical ventilation (EMA, 2020). A preliminary report shows that it can lower the 28‐day mortality in this patient group (EMA, 2020; Horby et al., 2020). Additionally, FDA has authorized the monoclonal antibody therapies casirivimab and imdevimab for treatment of mild to moderate COVID‐19 (FDA, 2020).

Since it has been shown that COVID‐19 increased the number of thrombotic events, heparin has been used to prevent these events. A study conducted in Spain showed that heparin correlated with a lower mortality (OR = 0.55 (0.37, 0.82), *p* = 0.003). Nevertheless, the evidence of this association is still limited, and further research is needed (Ayerbe et al., 2020).

Human recombinant soluble ACE2 (hrsACE2) is a potential candidate for treatment of COVID‐19. Using human blood vessels and kidney organoids, it has been shown to significantly inhibit SARS‐Cov‐2 infection in vitro (Monteil et al., 2020). Zoufaly el al. also reported that they saw promising results when they successfully treated a 45‐year‐old woman with severe COVID‐19 with hrsACE2 therapy. The drug binds the viral S protein and thus neutralises SARS‐CoV‐2 and inhibits viral invasion into the host cells. Moreover, it converts ANG‐II to ANG‐(1‐7) and ANG‐I to ANG(1‐9), thus upregulating the ACE2/ANG‐(1‐7)/MAS‐axis (Zoufaly et al., 2020). Furthermore, due to downregulation of membrane‐bound ACE2 in COVID‐19 patients (Banu et al., 2020) and thus downregulation of the ACE2/ANG‐(1‐7)/MAS‐axis, ANG‐(1‐7)‐analogues are targets for therapy. Treatment of ANG‐(1‐7)‐analogues could stimulate the anti‐inflammatory and antifibrotic effects of the ACE2/ANG‐(1‐7)/MAS‐axis. ANG‐(1‐7)‐analogues are currently tested in clinical trials (NCT04332666, NCT04401423).

Another candidate for drug targeting is the viral main protease M^pro^, which plays a vital role in viral replication. Dai et al. and Ma et al. have developed inhibitors of M^pro^ which have shown promising results in vitro (Dai et al., 2020; Ma et al., 2020). Table 3 gives an overview of clinical studies involving RAAS‐directed COVID‐19 therapy approaches.

**TABLE 3:** ANG‐(1‐7) and analogues currently tested in clinical trials

Title (study)	Purpose	Study design	Methods	Status	Phase
*Angiotensin‐(1,7) treatment in COVID‐19: the ATCO trial* **(NCT04332666)**	Evaluating the efficacy and clinical effects of treatment with ANG‐(1‐7) in patients with COVID‐19 requiring mechanical ventilation	Randomised, controlled, adaptive Phase II/III trial, single‐blinded interventional trial	60 participants with an age of minimum 18 years with COVID‐19 requiring mechanical ventilation The study consists of two phases: Phase II and III Phase II includes 20 persons. The purpose of this phase is to confirm the safety of the drug. The purpose of Phase III is to evaluate the efficacy and clinical impact of the drug In both phases, one group of 30 participants received 0.2 mcg/kg/h of the drug, intravenously, for 48 hours. The other group (30 participants) received a placebo‐drug Primary outcome: number of ventilator‐free days at day 28 Secondary outcomes: ICU free days, hospital length of stay, time to wean from mechanical ventilation, PaO2/FiO2 changes during administration of drug, incidence of deep vein thrombosis, changes in inflammatory markers, plasma levels of ANG‐II and ANG‐(1‐7), CT scan changes	Not yet recruiting	2/3
*Randomized controlled trial of angiotensin 1*‐*7 (TXA127) for the treatment of severe COVID*‐*19* **(NCT04401423)**	Determining if treatment of TXA127 prevents AKI and multi‐organ failure in patients with severe COVID‐19	Double‐blinded, placebo‐control, randomised clinical trial	100 participants with an age of minimum 18 years with severe COVID‐19 One group receives one three‐hour dosage of TXA127 (0.5 mg/kg, intravenously) for 10 consecutive days. Another group receives one three‐hour dosage of a placebo‐drug (0.5 mg/kg, intravenously) for 10 consecutive days Primary outcome: change of serum creatinine and the number of patients requiring intubation and ventilatory support Secondary outcome: change in the number of deceased participants, number of participants requiring dialysis and vasopressors, change in blood inflammatory markers, percent change in supplemental oxygen requirements	Recruiting	2
*Angiotensin (1*‐*7) for the treatment of COVID*‐*19 in hospitalised patients* (NCT04570501)	Evaluating the effects of intravenous (IV) treatment of ANG‐(1‐7) for treatment of COVID‐19	Randomised, double‐blind, placebo‐controlled study.	160 participants with an age of minimum 18 years hospitalised with COVID‐19 Participants are divided into two groups. One group receives ANG‐(1‐7) treatment IV for 7 days. The other group receives a placebo drug IV for 7 days Primary outcome: time to recovery	Not yet recruiting	½
*Evaluation of the possible role of angiotensin peptide (1*‐*7) on treatment* of COVID‐19 (NCT04375124)	Evaluate the effects of plasma‐derived ANG‐(1‐7) supplementation in the treatment of COVID‐19	Interventional	20 participants (≥18 years) infected with SARS‐CoV‐2 The study population are divided into two groups. One group receives routine treatment for COVID‐19. As well as receiving routine treatment for COVID‐19, the other group receives ANG‐(1‐7) supplement Primary outcome: mortality over the period of 4 months	Recruiting	Not applicable
*Angiotensin 1‐7 as a therapy for pneumonia caused by Coronavirus 2(SARS‐CoV‐2)* (NCT04605887)	Treatment of COVID‐19	Double blind, randomised study	120 participants (18 years or older) with SARS‐CoV‐2 infection, fever (>37.8 ℃), cough and dyspnea 60 participants receive placebo once daily. 60 participants receive ANG‐(1‐7) subcutaneously (500 mcg/kg/day). The participants are treated in 14 days or until they are discharged from hospital Primary outcome: the need for mechanical ventilation, death	Not yet recruiting	2
*Randomised clinical trial phase I/II for the use of angiotensin‐(1‐7) in the treatment of severe infection by Sars‐CoV‐2* (NCT04633772)	Determining the effects of IV ANG‐(1‐7) treatment for patients in the intensive care unit (ICU) with severe COVID‐19	Randomised controlled trial	130 participants (17 to 81 years) who have been admitted to ICU with severe pneumonia The study consists of two phases. Phase I includes 30 participants with the purpose to evaluate the safety of IV infusion of the drug Phase II includes 100 participants who are divided into two groups. One group receives ANG‐(1‐7) IV. The other group receives a placebo drug. The duration is 28 days Primary outcome: number of supplemental oxygen‐free days. Secondary outcomes: length of stay in hospital, ventilator‐free days, ICU free days, RAAS components levels, CT scan findings, changes in clinical state, chest X‐ray findings, changes in C‐reactive protein, chemokines, troponin, troponin, and D‐dimer	Recruiting	½

Currently, the availability of prophylaxis for COVID‐19 is limited, but several vaccines are in preclinical and clinical trials worldwide. One vaccine has been approved for use in the Chinese military (Poland et al., 2020). The mRNA vaccine Gam‐COVID‐Vac (Sputnik V) has controversially also been approved for use in Russia (Callaway, 2020; Logunov et al., 2020). Pfizer and BioNTech announced the first results from a Phase 3 clinical trial of the vaccine candidate BNT162b2. The results show an efficacy rate above 90% in participants receiving a second dose of the vaccine (Polack et al., 2020). The vaccine has been approved for use in the United Kingdom, where vaccinations started on 8 December, 2020 (Hancock, 2020), as well as in the EU. Yet, persons with a history of a serious allergic reaction to a vaccine should not receive the vaccine, since there have been cases of anaphylactic reactions (Mahase, 2020). Furthermore, the Moderna and Astra Zeneca vaccines were also approved by EMA and FDA. Examples of COVID‐19 vaccine candidates are listed in Table 4.

**TABLE 4:** Selected COVID‐19 vaccine candidates

Company	Vaccine	Type	Stage/Authorization
BioNTech and Pfizer	BNT162b2	mRNA based	Phase III, authorized in EU, US, UK, Canada, Israel, Switzerland, Norway, Saudi Arabia, WHO‐validated
Moderna Biotech Spain	mRNA‐273	mRNA based	Phase III, authorized in EU, US, UK, Canadam Israel, Norway, Canada, Saudi Arabia, Switzerland
Astra Zeneca and Oxford University	ChAdOx1‐SARS‐CoV‐2	Vector virus‐based	Phase III, authorized in EU, UK, Saudi Arabia, Brazil,
Sputnik V	Gam‐COVID‐Vac	Vector‐virus‐based	Phase III, athorized in Russia, Hungary, UAE, Argentina, Turkmenistan, Iran, Pakistan, Uruguay, Venezuela
Sinovac	CoronaVac	Inactivated SARS‑CoV‑2	Phase III, authorized in China, Turkey, Brazil, Indonesia, Uruguay, Chile, Colombia, Bolivia
Sinopharm	BBIBP‐CorV	Inactivated SARS‑CoV‑2	Phase III, authorized in China, Bahrain, UAE, Seychelles, Egypt, Hungary, Pakistan,
Curevac	Zorecimeran	mRNA‐based	Phase III
Janssen‐Cilag International N.V.	Ad26.COV2.S	Vector virus‐based	Phase III, authorization pending in EU (Johnson & Johnson requested EU vaccine authorization on 2/16/2021), approved in US, Canada and South Africa
Novavax	NVX‐CoV2373	Subunit vaccine	Phase III, authorization pending in EU, Canada

Modified from (EMA, 2020).

### 5.1 COVID‐19: Effects on the RAAS and Hypertension

A meta‐analysis conducted by Zhang et al. showed an association between HT and COVID‐19 severity and fatality. In patient groups with an age of <50 years and ≥50 years, the risk of severe HT was increased by a factor of 2.21 and 2.23, respectively. Additionally, the analysis showed that hypertensive patients have a 3.48‐fold higher risk of fatality compared to nonhypertensive patients (Zhang, Wu, et al., 2020).

Another meta‐analysis by Pranata et al. included 6560 patients with COVID‐19 pneumonia from 30 studies. The analysis showed that hypertension was associated with an increased severity of COVID‐19 pneumonia (RR = 2.04 (1.69, 2.47), *p* <0.001) and increased mortality (RR 2.21 (1.74, 2.81), *p* <0.001) (Pranata et al., 2020).

The studies imply that HT is a risk factor for severe or fatal COVID‐19. The mechanism of HT in relation to severe COVID‐19 is currently unclear. Since the virus enters the host cells by interacting with ACE2, it is hypothesised that the endocytosis of the virus‐ACE2 complex reduces the function of ACE2, leading to an overactivation of the ACE/ANG‐II/AT_1_R‐axis and resulting in complications such as ARDS and multiorgan failure (Zhang, Wu, et al., 2020).

Kuba et al. conducted an in vivo study on mouse lungs and showed a reduction of the ACE2 expression as a complication of the SARS‐CoV infection. Besides, by blocking the RAAS pathway, they saw an attenuation of the effects of the SARS‐CoV on the lungs (Kuba et al., 2005).

Moreover, elevated levels of plasma‐ANG‐II were observed in patients suffering from COVID‐19 (Liu et al., 2020). Plasma‐ANG‐II was linearly associated with viral load and lung injury (Liu et al., 2020). Additionally, studies have shown that patients with pulmonary arterial hypertension exhibited reduced ACE2 activity (Hemnes et al., 2018). These studies indicate that SARS‐CoV‐2 downregulates the expression of ACE2 on tissue, which leads to overactivity of the ACE/ANG‐II/AT_1_R‐axis and inhibition of the counterregulatory ACE2/ANG‐(1‐7)/MAS axis, resulting in lung injury. This can also explain why hypertensive patients are more prone to severe or fatal COVID‐19 infections.

One of the first‐line medications in the treatment of HT are the blockers of the RAAS, ARBs and ACEIs (Williams et al., 2018). The effects of ARBs and ACEIs in relation to COVID‐19 are highly debated. ACEI and ARBs induce an increase in ACE2 expression in cardiac tissue, and several tissues in rodents including cardiac and renal tissue. Therefore, some authors hypothesise that these drugs increase the risk of the SARS‐CoV‐2 infection. However, there is no significant evidence supporting the fact that ARBs and ACEIs increase the risk and severity of COVID‐19 (Ferrario et al., 2005; Kreutz et al., 2020; Rico‐Mesa et al., 2020). Furthermore, randomized clinical trials comparing the effects discontinuing vs. continuing ACEIs and ARBs therapy have found no evidence for discontinuing ARBs and ACEIs in patients with COVID‐19 (Cohen et al. 2020; Lopes et al., 2021). On the contrary, it is more likely that the discontinuation of antihypertensive treatment increases the severity of COVID‐19. Since the dysregulation of RAAS is a major factor in lung injury in COVID‐19, the drugs might decrease the severity of symptoms by balancing the two axes of the RAAS. Zhang et al. conducted a retrospective study with 1128 HT‐patients (ARB/ACEI‐users and non‐ARB/ACEI‐users) infected with COVID‐19. They found a significantly lower rate of mortality in the group with ARB/ACEI‐users compared to the group of non‐ARB/ACEI‐users (adjusted HR = 0.42, *p* = 0.03). Likewise, they reported a lower incidence of septic shock and disseminated intravascular coagulation when comparing the two study groups (Zhang, Zhu, et al., 2020). Supporting this, Greco et al. performed a meta‐analysis including 14 studies finding no increase in severe or fatal COVID‐19 associated with an ARB or ACEI therapy (Greco et al., 2020). These studies illustrate the fact that rather than having a negative effect, the ARBs/ACEIs may have a protective effect in relation to the severity and fatality of COVID‐19 in HT patients. Further studies are needed to determine the mechanisms behind the effect of the drugs in relation to COVID‐19. The association between ARBs/ACEIs and risk of SARS‐CoV‐2 infection is yet to be determined.

## 6 DISCUSSION

The COVID‐19 pandemic is currently causing major health issues with a high rate of hospital admissions and deaths worldwide. Many patients are admitted to the hospital for a longer time and need an extended time to recover. Even patients who recovered from a moderate infection reported weakness, tiredness, and problems to smell and/or taste (Carfì et al., 2020; Rees et al., 2020; Tian et al., 2020). A recent meta‐analysis also hinted towards a possible connection between COVID‐19 and new‐onset diabetes, showing that 14.4% of hospitalized COVID‐19 patents also suffered from newly diagnosed diabetes (Sathish et al., 2020).

Besides complications such as ARDS and AKI, SARS‐CoV‐2 also leads to cardiovascular damage (Craver et al., 2020; Kim et al., 2020; Lu et al., 2020; Naneishvili et al., 2020). Studies have shown the presence of SARS‐CoV‐2 genomes in cardiac tissue (Lindner et al., 2020). Furthermore, in endomyocardial biopsies permeation of small vascular walls was observed, and this is hypothesised to cause myocardial ischemia leading to arrythmias (Escher et al., 2020). SARS‐CoV‐2 has also been found within endothelial cells causing an endotheliitis, which is hypothesised to be the reason for the systemic complications of COVID‐19. Therefore, stabilisation of vascular endothelium in combination with antiviral treatment could be an effective way of treating COVID‐19 (Varga et al., 2020).

It is obvious that HT is a major risk factor regarding the severity and fatality of COVID‐19. However, it should be noted that the studies conducted by Zhang et al. and Pranata et al. exhibit some limitations. One limitation is the fact that the included studies can have different definitions of HT, leading to an overestimation or underestimation of the relation between HT and risk of severe COVID‐19. Pranata et al. did also include many studies yet to be peer‐reviewed. Lastly, information about the effect of antihypertensive medication could not be estimated, which underestimates the relation between HT and severity of COVID‐19 (Pranata et al., 2020; Zhang, Wu, et al., 2020).

Supporting the fact that HT increases the severity of COVID‐19, it has been shown that treatment with ARBs or ACEIs decreases the risk of death in HT‐patients with COVID‐19 (Zhang, Zhu, et al., 2020). However, the number of patients receiving ARBs/ACEI in this study was only 188 which can cause statistical bias. Furthermore, the study is a retrospective study and not all data about the patients’ medication could be retrieved (Zhang, Zhu, et al., 2020). However, a cohort study by Yan et al. illustrated a decreased fatality of COVID‐19 patients with HT using ARBs. Weaknesses of this study are that the cohort is small (655 patients) and the majority were old patients (>65 years) (Yan et al., 2020). Hence, the study indicates protective effects of ARBs/ACEI regarding the treatment of hypertensive patients with severe COVID‐19, but further studies are needed to confirm these effects.

Most patients with severe COVID‐19 have comorbidities, for instance, HT and diabetes, and they tend to be older (Du et al., 2020; Wang, Yin, et al., 2020; Zheng et al., 2020; Zhou et al., 2020). These patients are associated with ACE2 deficiency. Since studies indicate that ACE2 expression is reduced because of SARS‐CoV‐2 infection, this effect added to the ACE2 deficiency might worsen the dysregulation of the RAAS. This could explain the fact that patients with the mentioned features are more prone to severe COVID‐19 (Verdecchia et al., 2020). Nonetheless, further studies are needed to clarify exactly why patients with features such as HT, diabetes and old age are more likely to have severe complications of COVID‐19. Additionally, the mechanism of HT regarding severe COVID‐19 should be determined.

An infection of SARS‐CoV‐2 is associated with the decreased expression of ACE2 and elevated levels of plasma‐ANG‐II, indicating dysregulation of the two arms of RAAS (Kuba et al., 2005; Liu et al., 2020). Therefore, a potential target of COVID‐19 treatment is increasing the plasma‐levels of ACE2 through exogenous ACE2‐therapy and thereby upregulating the ACE2/ANG‐(1‐7)/MAS‐axis and downregulating the opposing axis of RAAS. Although there exists only a limited number of cases being treated with hrsACE2, the patients being treated have shown great results reinforcing the idea of future treatment of COVID‐19 with exogenous ACE2 (Zoufaly et al., 2020).

This review has some limitations. Firstly, there is publication bias since the references used the work were only in English, thus studies in other languages may have been excluded. Lastly, since the disease is relatively new, and most of the study population are Chinese, the geographic diversity is limited.

## 7 CONCLUSION AND OUTLOOK

HT is a serious health problem worldwide, and studies have shown that it increases the severity and fatality of COVID‐19. It has been demonstrated that RAAS plays a pivotal role in COVID‐19 infection. It is known that an infection of SARS‐CoV‐2 can lead to a dysregulation of the RAAS by reducing the expression of ACE2. Although SARS‐CoV‐2 has emerged recently, the current treatments and the prophylaxis of COVID‐19 is limited, although several drugs/vaccines as candidates for treatment/prophylaxis of COVID‐19 have been tested in clinical trials. The research field is developing rapidly, and almost every day, new information and data about COVID‐19 are published, such as a report on a SARS‐CoV‐2 blocker, which has fully prevented infection of cells in vitro (Svilenov et al., 2020) or on a ribonucleoside analogue, which was able to suppress SARS‐CoV‐2 transmission in ferrets (Cox et al., 2021).

The exact mechanisms of HT in relation to COVID‐19, and the biological processes behind the ACE2 downregulation due to SARS‐CoV‐2 remain uncertain, and further research is needed to determine these mechanisms.

## CONFLICTS OF INTEREST

The authors declare no conflict of interests.

## AUTHOR CONTRIBUTION

Briyanth Ravichandran, Daniela Grimm and Markus Wehland designed the present review. Briyanth Ravichandran and Marcus Krüger designed the figures. Briyanth Ravichandran, Daniela Grimm and Markus Wehland wrote the manuscript. All authors analyzed data and references. All authors read and approved the final manuscript.
